# Negative Impact of Wound Complications on Oncologic Outcome of Soft Tissue Sarcomas of the Chest Wall

**DOI:** 10.3390/cancers12010101

**Published:** 2019-12-31

**Authors:** Mehran Dadras, Pascal Koepp, Johannes Maximilian Wagner, Christoph Wallner, Maxi Sacher, Marcus Lehnhardt, Björn Behr, Kamran Harati

**Affiliations:** Department of Plastic Surgery, BG University Hospital Bergmannsheil, 44787 Bochum, Germany

**Keywords:** soft tissue sarcoma, wound complications, tumor, chest wall

## Abstract

A link of complications with worse oncologic prognosis has been established for multiple malignancies, while the limited literature on soft-tissue sarcomas is inconclusive. The aim of this study was to examine risk factors and the oncologic impact of wound complications after curative resection of primary soft-tissue sarcomas of the chest wall. Patients with primary soft tissue sarcomas of the chest wall were identified. Groups with and without wound complications were compared by using univariate and multivariate analysis to identify risk factors. For patients with clear surgical margins (R0), univariate and multivariate analysis of factors associated with 5-year local recurrence free survival (LRFS), metastasis free survival (MFS), and disease specific survival (DSS) were performed. A total of 102 patients were included in the study. Wound complications occurred in 11 patients (10.8%) within 90 days. Cardiovascular morbidity and operation time represented independent risk factors for wound complications. In 94 patients with clear surgical margins, those with wound complications had an estimated 5-year LRFS of 30% versus 72.6% and a 5-year DSS of 58.3% versus 82.1%. Wound complications could be identified as an independent predictor for worse LRFS and DSS. Patients with a high risk of wound complications should be identified and strategies implemented to reduce surgical complications and possibly improve oncologic prognosis.

## 1. Introduction

Soft tissue sarcomas (STS) represent a heterogeneous group of malignant tumors with mesenchymal origin and represent around 1% of all adult malignancies [1]. Of these, approximately 10% arise from the chest wall [1,2].

Surgical resection is considered to be the cornerstone of treatment, while complete resection offers the potential for curative treatment.

Among the factors that are predictive for oncologic outcome, surgical margins, histological grade and subtype, tumor size, and depth are considered to be the most significant [2,3,4,5,6].

Multiple studies have addressed the impact of multimodal therapy on the treatment of STS. While radiotherapy has been shown to improve local control, the timing of therapy as adjuvant or neoadjuvant has been a subject for debate. It could be observed that neoadjuvant radiotherapy increases the risk of wound complications, whereas more fibrosis has been observed in adjuvant application [7,8,9]. According to German guidelines, radiotherapy of soft tissue sarcomas is recommended in high-grade lesions and tumors >5 cm in maximum diameter [10,11], but use of multimodal therapy ultimately remains a case-by-case decision based on individual factors.

Local inflammatory complications have been identified as a predictor for cancer recurrence and long-term oncologic outcome in various malignancies. An association of anastomotic leak with higher local recurrence rates has been shown for colorectal, esophagus, and gastric cancer [12,13,14]. Similarly, wound complications have been shown to be a negative predictor for oncologic outcome of squamous cell carcinoma of the head and neck [15,16] or breast cancer [17,18].

So far, only two studies have addressed the impact of surgical complications on oncologic outcome of STS to our knowledge [19,20]. Both studies included patients with STS in different locations, incomplete resections, and significant differences in the application of multimodal therapy of the compared subgroups, representing possible biases for the results.

Thus, the aim of this study was to assess the oncological impact of surgical complications in a tightly defined patient population such as curative resections of primary STS of the chest wall.

Secondarily, predictors of surgical complications in this cohort of patients should be identified.

## 2. Results

A total of 144 patients with STS of the chest wall were treated at our institution between January 1995 and December 2016. After excluding 38 patients who initially presented with a sarcoma recurrence, two patients with metastatic disease upon diagnosis, and two patients with missing relevant data, the remaining 102 patients were included in the study. Sexes were equally distributed with 51 patients each. Median age upon resection of the tumor was 58 years. 56 patients presented at our institution primarily, while 46 patients had undergone prior external biopsy or insufficient operative treatment. The most prevalent histologic subtypes were undifferentiated pleomorphic sarcoma in 24 cases, liposarcoma in 17 cases, and angiosarcoma in 10 cases. The most common tumor locations were anterolateral thorax in 32 cases and lateral thorax in 28 cases, while 71 tumors were located below the deep fascia. In terms of size, 69 tumors were larger than 5 cm and the mean largest diameter was 9 ± 9 cm. Furthermore, 34 tumors were graded as G1, 29 as G2, and 39 as G3.

Full thickness resection of the thoracic wall was needed in 39 patients.

Soft tissue closure could be achieved primarily in 70 patients, seven patients received skin grafting, 24 patients received local grafting, and one patient received free microvascular flap coverage.

Negative margins could be achieved in 94 of patients, while microscopical (R1) incomplete resection was performed in four cases and macroscopical incomplete resection (R2) was performed in four cases.

Five patients received neoadjuvant therapy, as all of these patients had a high grade (G2/G3) tumor. Out of these patients, one received neoadjuvant radiotherapy, two received chemotherapy, and two received combined radiochemotherapy.

Adjuvant radiotherapy was administered to 33 patients with a mean dose of 56 ± 6 Gray. Out of these patients, 31 had a high grade (G2/G3) tumor.

During the 90-days postoperative period, 45 patients developed complications as presented in Table 1. A total of 20 of these complications were classified as Clavien-Dindo (CD) grade 1 and hence as being very minor. Meanwhile, 25 patients developed a CD grade ≥2 complication. Wound infections occurred in 13 patients, nine of which underwent operative treatment. Only CD grade ≥2 overall and wound complications were considered for further analysis.

Clavien Dindo classification: Grade I: Any deviation from the normal postoperative course without the need for pharmacological treatment except for a number of allowed substance categories or surgical, endoscopic, and radiological interventions. Grade II: Requiring pharmacological treatment with drugs other than the ones allowed for grade I. Grade III: Requiring surgical, endoscopic or radiological intervention (a: under local anesthesia; b: under general anesthesia). Grade IV: A life-threatening complication requiring intensive care treatment (a: single organ; b: multi- organ dysfunction). Grade V: death.

### 2.1. Predictors of Wound Complications

A comparison of groups with and without complications is presented in Table 2. A significantly higher proportion of obese patients developed wound complications, however obesity was eliminated in multivariate analysis. Both the ASA-score and cardiovascular morbidity were significantly higher in the wound complication group, in multivariate analysis the ASA-score was eliminated by cardiovascular morbidity, which represented an independent predictor of wound complications with an odds ratio of 5.07 (1.11–23.16) and *p* = 0.036.

Neither tumor depth, location, nor size showed significant differences in the patient group with and without wound complications, yet the wound complication rate of tumors <5 cm was 6% compared to 23% in tumors >15 cm. Angiosarcomas had the highest wound complication rate with 30%, while the distribution of histologic types of sarcoma did not differ significantly overall. No association of wound complications was identified with the stage of disease, tumor grade, or resection status but operation time represented an independent predictor of wound complications with an odds ratio of 1.01 (1.001–1.02) per minute, *p* = 0.025. Patients with complex soft tissue coverage did not have a higher wound complication rate.

### 2.2. Follow-Up

The median follow-up of surviving patients was 10.1 years, and 73.5% of surviving patients had a follow-up after at least 60 months.

A total of 36 patients (34.5%) developed a recurrence during the follow-up period, the 5-year LRFS was 67.5%, and the 10-year LRFS was 61.2%. Meanwhile, 22 patients (21.2%) developed metastases, the 5-year MFS was 78.3%, and the 10-year MFS was 71.9%. Among patients with recurrence, 20 had a single recurrence, nine had two recurrences and seven had three or more recurrences. A total of 35 patients (33.7%) died during the follow-up period, resulting in a 5-year Overall survival (OS) of 72.7% and a 10 year-OS of 61.2%. A total of 26 deaths were disease specific, resulting in a 5-year DSS of 73.5% and a 10-year DSS of 70.9%.

A total of eight patients with incomplete resections (R1, n = 4 and R2, n = 4) were excluded from analysis of predictors of outcome. Univariate analysis results of predictors of 5-LRFS, MFS, and DSS are presented in Table 3, along with multivariate analysis in Table 4.

### 2.3. Local Recurrence Free Survival (LRFS)

**Figure 1:**
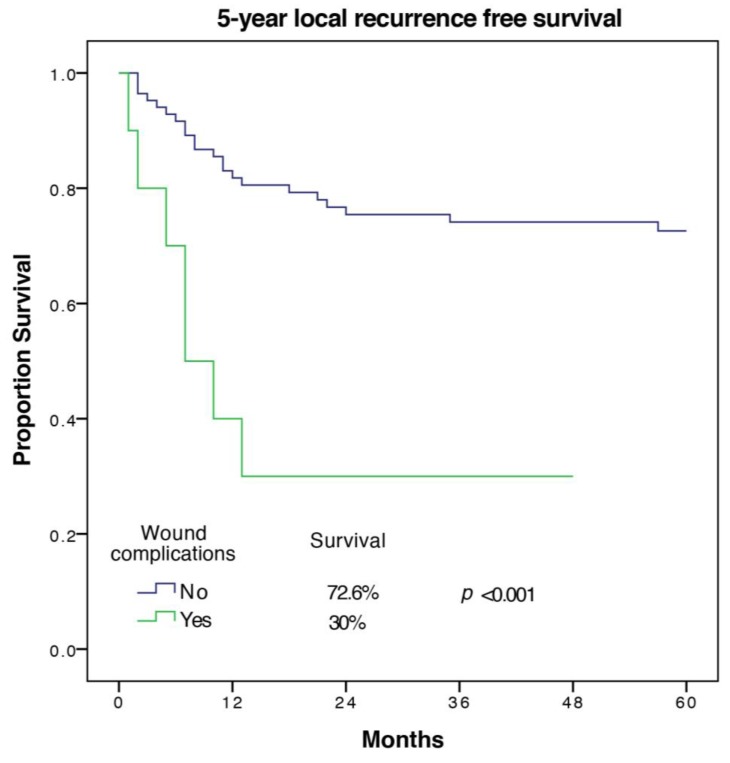
5-year local recurrence free survival of patients with and without wound complications.

A total of 29 out of the remaining 94 patients (30.9%) developed a local recurrence within 5 years. In univariate analysis of 5-year LRFS, female sex, a high ASA-score, and cardiovascular morbidity had a negative impact, but only cardiovascular morbidity was confirmed as a predictive factor in multivariate analysis with a hazard ratio of 4.07 (1.43–11.59) and *p* = 0.009. Histopathologic subtype, tumor size, UICC-stage, and tumor grade were predictors of LRFS in univariate analysis. Tumor grade remained an independent predictor in multivariate analysis while the UICC-stage was not included in the model. Wound complications with a CD grade of ≥2 were found to be significantly associated with worse LRFS in univariate and multivariate analysis with a hazard ratio of 3.83 (1.24–11.85), *p* = 0.02, and a 5-year LRFS of 30 ± 14.5% versus 72.6 ± 5%. The Kaplan Meier 5-year LRFS curve for wound complications is shown in Figure 1.

Adjuvant radiation was significantly associated with higher LRFS, both in univariate and multivariate analysis and had a HR of 0.09 (0.03–0.31), *p* < 0.001.

### 2.4. Metastasis Free Survival (MFS)

**Figure 2:**
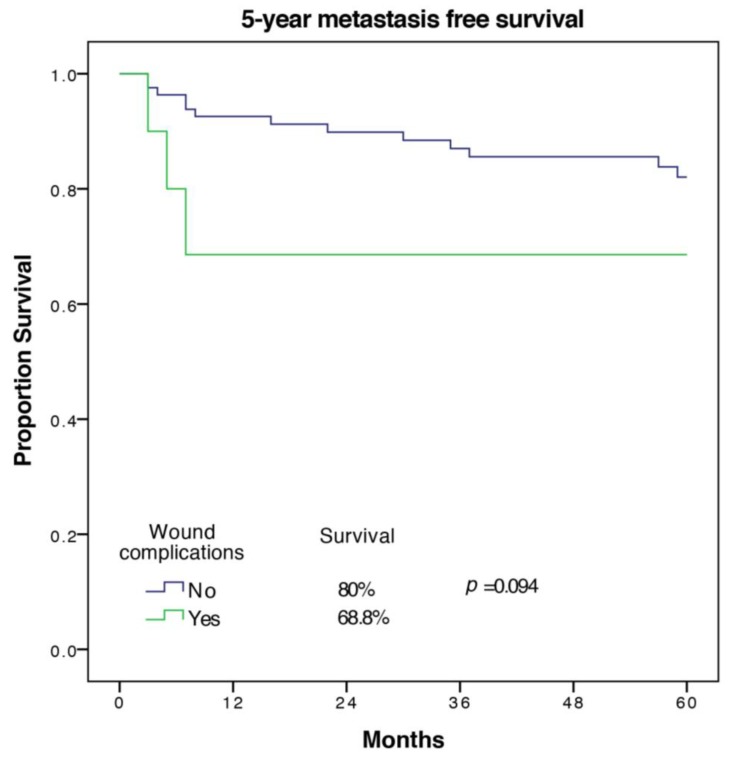
5-year metastasis free survival of patients with and without wound complications.

A total of 16 out of 94 patients (17%) developed metastases in the first five years. Factors associated with worse MFS on univariate analysis were age (*p* = 0.035), cardiovascular morbidity (*p* = 0.011), tumor size (*p* = 0.003), UICC-stage (*p* = 0.022), and tumor grade (*p* = 0.015). The only significant predictor of MFS on multivariate analysis with exclusion of UICC-stage was tumor grade (HR 14.35 (1.31–157.35), *p* = 0.029 for G3 vs. G1). Wound complications were associated with a trend towards a lower MFS (68.8 ± 15.1% versus 82 ± 4.6%) but did not meet statistical significance in univariate analysis (*p* = 0.095). The Kaplan Meier 5-year MFS curve for wound complications is shown in Figure 2.

### 2.5. Disease Specific Survival (DSS)

**Figure 3:**
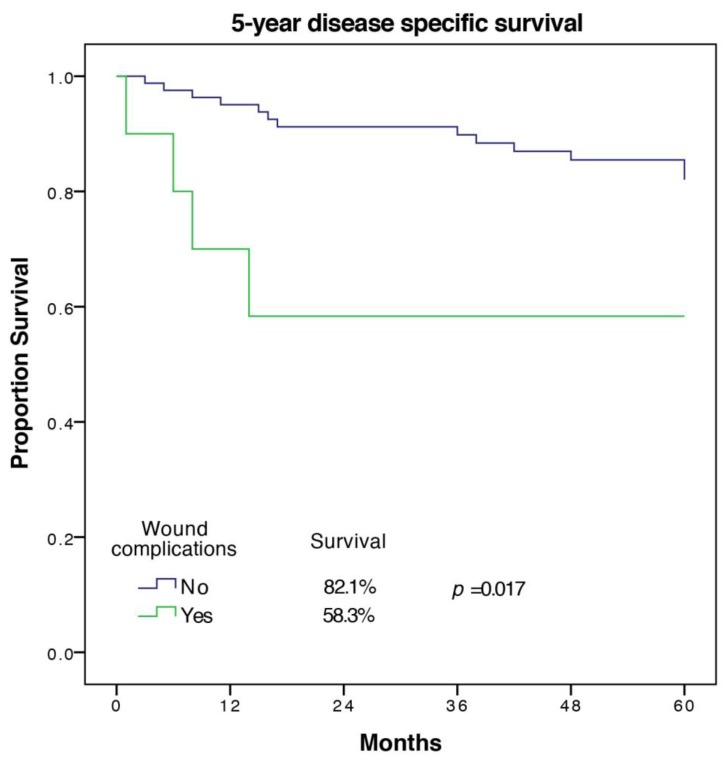
5-year disease specific survival of patients with and without wound complications.

A total of 17 out of 94 patients (18%) died from the underlying disease in the first five years. Factors associated with worse DSS on univariate analysis were age (*p* = 0.023), cardiovascular morbidity (*p* = 0.001), tumor size (*p* = 0.019), UICC-stage (p = 0.046), tumor grade (*p* = 0.045), wound complication (*p* = 0.017), and other complication (*p* = 0.051). Significant predictors of DSS in multivariate analysis with the exclusion of the UICC-stage were cardiovascular morbidity (HR 11.55 (2.23–59.82), *p* = 0.004), as well as tumor grade and wound complications (HR 5.31 (1.1–25.62), *p* = 0.037). The Kaplan Meier 5-year DSS curve for wound complications is shown in Figure 3.

## 3. Discussion

In the present study, postoperative complications after a resection of 102 primary STS of the chest wall were analyzed and predictors of wound complications were identified. In 94 patients with achieved complete resection (R0), predictors for LRFS, MFS, and DSS were analyzed to examine the impact of wound complications on oncologic outcome.

Most tumors were high grade and larger than 5 cm in diameter, as in comparable studies in the literature [5,22,23,24]. The 5-year OS was 72% and 5-year LRFS was 67.5% with a median follow-up of 121 months. In a large cohort of 192, examination of the STS of the chest wall from Memorial Sloan Kettering Cancer Center with a median follow up of 51 months provided a 5-year LRFS of 69% and a 5-year OS of 73%, which was comparable to our results [22].

The rate of all complications including CD-grade 1 in our cohort of soft tissue sarcomas of the chest wall with 44% was comparable to a study of Slump et al. on STS of various locations that required flap coverage and Montgomery et al. on extremity STS [25,26].

Several studies have focused specifically on wound complications after surgical resection of STS and associated risk factors, although these cohorts mostly consist of extremity tumors [7,27,28,29].

In univariate analysis, obesity was associated with higher wound complications in our cohort. Accordingly, Montgomery et al. found obesity to be associated with both a larger tumor size and higher morbidity as causes of wound complications, and thus that it is eliminated by other factors in multivariate analysis [26].

Our data demonstrated that cardiovascular morbidity is a strong predictor of wound complications, which is in accordance with previous literature [25]. Operative time was also identified as an independent predictor of wound complications in our cohort and is a result of multiple factors such as tumor size, depth, complexity, morbidity of general anesthesia time, and possibly hypothermia. Analogously to other studies, patients who received skin grafting or flap coverage did not have higher wound complication rates [28,30].

In our analyses, we identified morbidity and tumor grade as predictors of oncologic outcome, which is in accordance with previous literature on truncal and extremity STS, albeit in other studies, age as a surrogate of morbidity was analyzed rather than morbidity itself [22,31,32].

Wound complications were identified as an independent predictor of local recurrence and disease specific death.

Only two studies have so far addressed the oncologic impact of complications in STS to our knowledge. Behnke et al. did not find a correlation of infectious wound complications with LRFS, MFS or DSS in 396 soft tissue sarcomas of the extremities with unreported median follow-up [19]. In contrast, Broecker et al. did find a significant impact of general complications on DSS in 546 patients with extremities and trunk STS after a median follow-up of 37 months, while influence on local recurrence was not significant [20].

Distribution of neoadjuvant radiation therapy differed significantly between groups and incomplete resections were included in both studies.

In our cohort of patients, less than 5% received neoadjuvant treatment and the proportion of patients who received adjuvant therapy did not differ substantially between groups with and without wound complications (27% vs. 35%). Only patients with microscopically clear surgical margins (R0) were included in the outcome analysis.

In a randomized controlled trial of neoadjuvant versus adjuvant radiotherapy for extremity STS, O’Sullivan et al. found no differences in LRFS despite a higher rate of wound complications in patients receiving neoadjuvant radiation therapy [8] In our series, only three patients received neoadjuvant therapy and thus no conclusions on the role of neoadjuvant therapy could be drawn. It is unclear whether a negative impact of wound complications exists only in STS without prior radiation. Further studies are needed to address this important question.

An association of local inflammatory complications with cancer recurrence has been demonstrated for multiple malignancies as discussed in the introduction of this article. It has been shown that most STS maintain an inflammatory microenvironment with an observed secretion of IL6, a proinflammatory cytokine [33]. Its use as a biomarker and therapeutic target has been the subject of studies [34,35]. In breast cancer, IL6 was able to activate dormant cancer cells, a mechanism that could also be relevant for sarcoma recurrence after local resection [36]. In a murine study, induced secretion of IL1β, one of the central mediators of acute infection, led to increased proliferation, migration, and invasion capacity of fibrosarcoma cells [37].

Therefore, although there are hints in the literature, the interaction between inflammation and STS deserves further investigations.

At the same time, inflammation has been shown to play an essential role in cardiovascular disease such as atherosclerosis [38]. As such, elevated inflammation markers such as C-reactive protein have been shown to be predictive of cardiovascular disease and cardiovascular events suggestive of ongoing mild systemic inflammation in these patients [39,40]. Interestingly, the Emerging Risk Factors Collaboration also found elevated inflammation markers to be predictive of death for several cancers, suggesting a clinical link between systemic inflammation and malignant disease [40]. This can partly explain our findings of cardiovascular morbidity being a risk factor for worse LRFS and DSS, potentially by increasing wound complications and continuously elevated levels of systematic inflammation.

The most identified risk factors for wound complications after STS resection in this or other studies are intrinsic to the patient (morbidity) or the tumor (size, operation time) and do not allow modification by the surgeon. However, they aid in the identification of patients at elevated risk for wound complications and adaption of surgical or perioperative treatment to this risk.

Patients who received complex soft tissue reconstruction after sarcoma resection did not have a higher rate of wound complications than patients with primary wound closure in this or previous studies [28,30]. For this reason, flap reconstruction should be considered with a low threshold whenever primary closure is regarded to be at risk. Ultimately, strategies to reduce wound complications and possibly improve oncologic outcome need to be developed and implemented.

Certain limitations of this study exist that are inherent to its retrospective nature and the sample size due to rarity of STS of the chest wall. While the causal correlation of wound complications with oncologic outcomes for STS cannot be established by our data, the previous experimental research cited here and similar associations in more prevalent malignancies support these findings.

## 4. Materials and Methods

The study was approved by the ethical committee of the Ruhr-University Bochum, approval number: 18-6469-BR. All of the patients who underwent resection of a STS of the thoracic trunk from January 1995 until December 2016 at our institution were identified. Only primary tumors without dissemination were included in the study.

Retrospectively, demographic, clinical and outcome data were obtained from the medical records. Tumors were classified by the AJCC/UICC 8th edition [41].

New follow-up data on recurrence, metastases, and survival were obtained prospectively by correspondence with patients or relatives. Complications occurring within 90 days of initial operation were identified and classified by the modified Clavien-Dindo classification, which is based on the therapy necessary to treat the complication [21]. A summary of the classification is presented in Table 1. Wound complications were defined as surgical site infections or wound dehiscence. According to the Clavien-Dindo classification, grade one wound complications received no intervention (or bedside wound opening), grade two typically received antibiotic treatment, and grade three wound complications needed operative revision.

Cardiovascular morbidity was defined by a preexisting diagnosis of cardiovascular disease and included coronary or peripheral artery disease.

The primary outcome was 5-year local recurrence free survival (LRFS), while the secondary outcomes were 5-year metastasis free survival (MFS) and 5-year disease specific survival (DSS). For outcome analysis, all patients with incomplete (R1,R2) resections or metastatic disease were excluded and only complications with a Clavien-Dindo grade of 2 or higher were considered.

LRFS was calculated from the date of resection until tumor recurrence or until the last follow-up in patients without recurrence. MFS was calculated from the date of resection until the occurrence of metastases or until the last follow-up in patients without metastasis. DSS was calculated from the date of resection until disease specific death or the last follow-up in patients without disease specific death. Here, the cause of death was assessed by correspondence with relatives and assumed in the presence of metastatic disease.

### 4.1. Treatment

Preoperatively, computed tomography scans and/or contrast-enhanced magnetic resonance imaging (MRI) of the chest and the tumor site were routinely performed. The surgical aim for all patients was complete resection of the primary tumor with negative margins. A lateral clear margin of 2 cm of healthy tissue was ensured whenever possible. In epifascial lesions, a deep clear margin of one fascial layer was intended. Full-thickness chest wall resections were performed on lesions infiltrating the ribs or intercostal space. Indications for adjuvant radiation or chemotherapy were given at the discretion of our interdisciplinary tumor board.

Following surgical treatment, the follow-up management for all patients included clinical examinations, chest X-rays, and contrast-enhanced MRIs every three months for the first two years, and then every six months for the next three years. The decision for whether follow-up MRIs and chest X-rays would be continued after five years was based on previous tumor behavior and the decision of the informed patient.

### 4.2. Statistics

SPSS Version 21 for Mac (IBM, Armonk, New York, USA) was used for all analysis. Data are presented as means ± standard deviation or median. Comparison of patients with and without wound complications was performed via chi-square or fisher exact test for categorical variables and the independent t-test for continuous variables. Subsequent multivariate regression analysis of factors with *p* < 0.01 with backwards elimination was performed to identify independent predictors. Hazard ratio was calculated and is presented as HR (95% CI).

For the patients included in the outcome analysis, survival analyses were conducted using Kaplan–Meier log-rank tests and multivariate Cox regression with the enter method, including all factors with *p* < 0.01 in log rank testing for at least one outcome measure except for the UICC-stage. *p* < 0.05 was considered as statistically significant.

## 5. Conclusions

We were able to identify wound complications as an independent predictor of local recurrence and disease specific death in our single institution cohort of primary STS of the chest wall. Patients at risk of wound complications after STS resection should be identified and strategies implemented to reduce surgical complications and possibly improve oncologic prognosis.

## Figures and Tables

**Table 1:** Postoperative complications by Clavien-Dindo grade.

Complication	Clavien-Dindo Grade [21]					
	I	II	IIIa	IIIb	IV	Total
Respiratory tract infection	–	1	–	–	1	2
Urinary tract infection	–	2	–	–	–	2
Hemorrhage/Anemia	8	5	–	2	2	17
Wound complication	2	2	–	9	–	13
Seroma formation	11	–	–	3	–	14
Pneumothorax	–	–	4	–	–	4
All Patients (highest grade complication)	20	7	4	11	3	45

**Table 2:** Population and comparison of groups with and without wound complications.

Variable	All Patients (n = 102)	No Wound Complications (n = 91)	Wound Complications (n = 11)	Comparison, *p*-value	Logistic Multivariate Regression, *p*-value	Odds Ratio (95% CI)
**Sex**				0.200		
Male	51 (50)	43 (47.3)	8 (72.7)			
Female	51 (50)	48 (52.7)	3 (27.3)			
**Age at operation, mean**	56.1 ± 18	56.7 ± 17,8	59.4 ± 20	0.508		
**Obesity (BMI>30 kg/m^2^)**				0.033	*	
Yes	21 (20.6)	16 (17.6)	5 (45.4)			
No	81 (79.4)	75 (82.4)	6 (54.5)			
**ASA-Classification**				0.005	*	
1	20 (19.6)	19 (20.9)	1 (9.1)			
2	58 (56.9)	55 (60.4)	3 (27.3)			
3	22 (21.6)	15 (16.5)	7 (63.6)			
4	2 (2)	2 (2.2)	0			
**Cardiovascular Morbidity**				0.026	0.036	5.07 (1.11–23.16)
Yes	41 (40.2)	33 (36.3)	8 (72.7)			
No	61 (59.8)	58 (63.7)	3 (27.3)			
**Active Smoker**				0.219		
Yes	82 (80.4)	75 (82.4)	7 (63.6)			
No	20 (19.6)	16 (17.6)	4 (36.4)			
**Tumor depth**				0.497		
Superficial	31 (30.4)	29 (31.9)	2 (18.2)			
Deep	71 (69.6)	62 (68.1)	9 (81.8)			
**Tumor location (thorax region)**				0.704		
Anterior	11 (10.8)	9 (9.9)	2 (18.2)			
Anterolateral	32 (31.4)	28 (30.8)	4 (26.4)			
Lateral	28 (27.5)	25 (27.5)	3 (27.3)			
Posterolateral	19 (18.6)	17 (18.7)	2 (18.2)			
Posterior	12 (11.8)	12 (13.2)	0			
**Tumor size**				0.389		
≤5 cm	34 (33.3)	32 (35.2)	2 (18.2)			
5.1–10 cm	40 (39.2)	36 (39.6)	4 (26.4)			
10.1–15 cm	15 (14.7)	13 (14.3)	2 (18.2)			
>15 cm	13 (12.7)	10 (11)	3 (27.3)			
**Histologic Subtype**				0.233		
Undifferentiated pleomorphic	24 (23.5)	21 (23.1)	3 (27.3)			
Liposarcoma	17 (16.7)	16 (17.6)	1 (9.1)			
Angiosarcoma	10 (9.8)	7 (7.7)	3 (27.3)			
Dermatofibrosarcoma	8 (7.8)	8 (8.8)	0			
Fibrosarcoma	7 (6.9)	5 (5.5)	2 (18.2)			
Leiomyosarcoma	7 (6.9)	7 (7.7)	0			
Myxofibrosarcoma	6 (5.9)	6 (6.6)	0			
Other	23 (22.6)	21 (23.1)	2			
**UICC-Stage**				0.138		
I	33 (32.4)	31 (34.1)	2 (18.2)			
II	13 (12.7)	13 (14.3)	0			
III	56 (54.9)	47 (51.6)	9 (81.8)			
**Tumor Grade**				0.425		
G1	34 (33.3)	32 (35.2)	2 (18.2)			
G2	29 (28.4)	26 (28.6)	3 (27.3)			
G3	39 (38.2)	33 (36.2)	6 (54.5)			
**Prior external biopsy/incomplete resection**				0.980		
Yes	46 (45.1)	41 (45.1)	5 (45.5)			
No	56 (54.9)	50 (54.9)	6 (54.5)			
**Resection status**				0.515		
R0	94 (92.2)	84 (92.3)	10 (90.1)			
R1	4 (3.9)	4 (4.4)	0			
R2	4 (3.9)	3 (3.3)	1 (9.9)			
**Operative time in minutes, mean**	111.1 ± 72.5	105,5 ± 69.2	158.1 ± 86	0.029	0.025	1.01 (1.001–1.02)
**Full thickness resection**				0.239		
Yes	39 (38.2)	33 (36.3)	6 (54.5)			
No	63 (61.8)	58 (63.7)	5 (45.5)			
**Soft tissue reconstruction**				0.279		
Primary Closure	70 (68.6)	64 (70.3)	6 (54.5)			
Skin graft	7 (6.9)	7 (7.7)	0			
Local flap	24 (23.5)	19 (20.9)	5 (45.5)			
Free flap	1 (1)	1 (1.1)	0			
**Neoadjuvant Treatment**				1.000		
Yes	5 (4.9)	5 (5.5)	0			
No	97 (95.1)	86 (94.5)	11 (100)			
**Adjuvant radiotherapy**				0.744		
Yes	35 (34.3)	32 (35.2)	3 (27.3)			
No	67 (65.7)	59 (64.8)	8 (72.7)			
**Length of stay in days, mean**	13.4 ± 10.4	11.4 ± 5.8	29.5 ± 21.6	<0.001		

Data reported as n (%) or mean ± standard deviation; *BMI* body mass index, *ASA* American Society of Anesthesiologists, *UICC* Union internationale contre le cancer, * variable was eliminated in stepwise regression.

**Table 3:** Univariate analysis of factors associated with 5-y LRFS, MFS, and DSS.

Variable	n	5-y-LRFS	*p*-value (Log Rank)	5-y-MFS	*p*-value (Log Rank)	5-y-DSS	*p*-value (Log Rank)
**All patients**	94	67.8 ± 5		80.3 ± 4.5		79.5 ± 4.5	
**Sex**			0.005		0.550		0.189
Male	45	81.4 ± 5.9		83.9 ± 5.6		84.9 ± 5.7	
Female	49	55.1 ± 7.4		76.1 ± 7.2		74.1 ± 6.8	
**Age at operation**			0.057		0.036		0.023
≤39	19	89.5 ± 7		94.7 ± 5.1		-	
40–64	38	68 ± 7.6		83.8 ± 6.8		77.6 ± 7.7	
≥65	37	55.3 ± 8.8		66.4 ± 9.1		69.0 ± 8.3	
**Obesity (BMI>30 kg/m^2^)**			0.732		0.874		0.336
Yes	18	72.2 ± 10.6		83.3 ± 8.8		88.9 ± 7.4	
No	76	66.7 ± 5.6		79.1 ± 5.3		76.8 ± 5.3	
**ASA-Classification**			0.009		0.208		0.238
1	20	95 ± 4.9		90 ± 6.7		89.7 ± 6.9	
2	51	62.4 ± 7.1		80.8 ± 6.2		78.3 ± 6.5	
3	21	52.4 ± 10.9		69.3 ± 11.9		75.9 ± 9.4	
4	2	-		50 ± 35.4		50 ± 35.4	
**Cardiovascular Morbidity**			0.005		0.011		0.001
Yes	36	48.9 ± 9.1		62.5 ± 10.3		59.7 ± 9.5	
No	58	78.6 ± 5.5		88.7 ± 4.4		90.5 ± 4.1	
**Active Smoker**			0.921		0.947		0.641
Yes	19	68.4 ± 10.7		81.5 ± 9.8		78.6 ± 9.5	
No	75	67.7 ± 5.6		80 ± 5.1		79.9 ± 5.1	
**Tumor depth**			0.150		0.064		0.158
Superficial	29	77.9 ± 8		91.3 ± 5.9		87.9 ± 6.6	
Deep	65	63.4 ± 6.1		75.4 ± 5.9		75.8 ± 5.7	
**Tumor location (thorax region)**			0.773		0.837		0.174
Anterior	11	72.7 ± 13.4		90.9 ± 8.7		90.9 ± 8.7	
Anterolateral	27	61.4 ± 9.7		80.9 ± 8.8		75.7 ± 8.7	
Lateral	26	67.2 ± 9.6		79.9 ± 8.1		64.6 ± 10.4	
Posterolateral	19	68.4 ± 10.7		80.7 ± 10.3		90.9 ± 8.7	
Posterior	11	80 ± 12.6		70 ± 14.5		88.9 ± 10.5	
**Tumor size**			0.001		0.003		0.019
≤5 cm	33	74.5 ± 7.9		81.9 ± 7.5		83 ± 7	
5.1–10 cm	35	76.2 ± 7.4		84.3 ± 6.5		83.1 ± 7	
10.1–15 cm	15	66.7 ± 12.2		92.9 ± 6.9		86.2 ± 9.1	
>15 cm	11	20.5 ± 12.9		46.7 ± 16.6		48.0 ± 16.4	
**Histologic Subtype**			0.013		0.321		0.96
Undifferentiated pleomorphic	23	59.1 ± 10.6		64.7 ± 11		66.9 ± 10.4	
Liposarcoma	14	92.3 ± 7.4		90.9 ± 8.7		90.9 ± 8.7	
Angiosarcoma	8	25 ± 15.3		66.7 ± 27.2		53.6 ± 20.1	
Dermatofibrosarcoma	8	-		-		-	
Fibrosarcoma	7	57.1 ± 18.7		85.7 ± 13.2		83.3 ± 15.2	
Leiomyosarcoma	6	83.3 ± 15.2		83.3 ± 15.2		66.7 ± 19.2	
Myxofibrosarcoma	5	60 ± 21.9		-		-	
Other	23	67.8 ± 10.1		76.4 ± 9.3		85 ± 8	
**UICC-Stage**			0.005		0.022		0.046
I	32	90.3 ± 5.3		96.7 ± 3.3		92 ± 5.4	
II	12	53.3 ± 16.1		59.5 ± 16.2		70.7 ± 14.6	
III	50	56.3 ± 7.2		74.5 ± 6.8		73.3 ± 6.7	
**Tumor Grade**			0.002		0.015		0.045
G1	33	90.6 ± 5.2		96.8 ± 3.2		92.4 ± 5.2	
G2	26	60.4 ± 9.8		75.6 ± 8.7		71.9 ± 9	
G3	35	51.4 ± 9		65.2 ± 9.9		72.3 ± 8.6	
**Prior external biopsy/incomplete resection**			0.306		0.704		0.728
Yes	42	73.1 ± 7		81.4 ± 6.4		76.6 ± 6.9	
No	52	63.3 ± 7		79.8 ± 6.2		82.9 ± 5.6	
**Full thickness resection**			0.101		0.916		0.146
Yes	36	59.6 ± 8.4		80.9 ± 7.2		73 ± 7.7	
No	58	72.9 ± 6		80 ± 5.8		83.4 ± 5.5	
**Soft tissue reconstruction**			0.305		0.159		0.673
Primary Closure	63	71.5 ± 5.9		80.2 ± 5.4		78.5 ± 5.6	
Skin graft	7	42.9 ± 18.7		83.3 ± 15.2		-	
Local flap	23	64.5 ± 10.1		86.5 ± 7.2		78 ± 8.7	
Free flap	1	-		-		-	
**Wound complication CD ≥ 2**			<0.001		0.094		0.017
Yes	10	30 ± 14.5		68.8 ± 15.1		58.3 ± 16.1	
No	84	72.6 ± 5		82 ± 4.6		82.1 ± 4.6	
**Other complication CD ≥ 2**			0.315		0.555		0.051
Yes	15	56.9 ± 13.4		77 ± 12		66 ± 12.4	
No	79	69.7 ± 5.3		80.9 ± 4.9		82.2 ± 4.7	
**Neoadjuvant Treatment**			0.761		0.385		0.723
Yes	4	75 ± 21.7		-		75 ± 21.7	
No	90	67.5 ± 5.1		79.5 ± 4.7		79.9 ± 4.6	
**Adjuvant radiotherapy**			0.036		0.114		0.762
Yes	30	81.7 ± 7.5		72 ± 8.5		81.3 ± 7.6	
No	64	61.2 ± 6.7		84.4 ± 5.2		78.6 ± 5.6	

*BMI* body mass index, *ASA* American Society of Anesthesiologists, *UICC* Union internationale contre le cancer. *5-y-LRFS* 5-year local recurrence free survival, *5-y-MFS* 5-year metastasis free survival, *5-y-DSS* 5-year disease free survival.

**Table 4:** Multivariate analysis of factors associated with 5-y LRFS, MFS, and DSS.

Variable	Hazard Ratio 5-y-LRFS	*p*-value (MVA)	Hazard Ratio 5-y-MFS	*p*-value (MVA)	Hazard Ratio 5-y-DSS	*p*-value (MVA)
**Sex**						
Male	0.93 (0.34–2.58)	0.893	2 (0.55–7.25)	0.289	1.75 (0.43–7.13)	0.432
Female	Ref.		Ref.		Ref.	
**Age at operation**						
≤39	Ref.		Ref.		Ref.	
40–64	1.73 (0.31–9.73)	0.533	1.27 (0.85–19.11)	0.861	–	0.935
≥65	1.62 (0.25–10.52)	0.612	3.26 (0.17–62.36)	0.433	–	0.935
**ASA-Classification**						
1	Ref.		Ref.		Ref.	
2	4.26 (0.44–41.2)	0.21	0.73 (0.09–5.9)	0.765	0.58 (0.07–4.6)	0.607
3	4.9 (0.47–50.87)	0.183	0.62 (0.07–5.85)	0.676	0.68 (0.08–5.38)	0.711
4	–	0.985	3.93 (0.13–122.81)	0.436	2.39 (0.1–55)	0.587
**Cardiovascular Morbidity**						
Yes	4.07 (1.43–11.59)	0.009	3.17 (0.72–13.91)	0.127	11.55 (2.23–59.82)	0.004
No	Ref.		Ref.		Ref.	
**Tumor depth**						
Superficial	Ref.		Ref.		Ref.	
Deep	1.46 (0.48–4.42)	0.508	1.88 (0.3–11.93)	0.503	1.5 (0.33–6.91)	0.600
**Tumor size**						
≤ 5 cm	Ref.		Ref.		Ref.	
5.1–10 cm	1.02 (0.33–3.16)	0.976	0.43 (0.09–2.13)	0.301	0.65 (0.13–3.12)	0.590
10.1–15 cm	0.94 (0.27–3.18)	0.916	0.08 (0.004–1.7)	0.105	0.52 (0.08–3.37)	0.496
> 15 cm	2.06 (0.63–6.74)	0.234	3.13 (0.59–16.64)	0.181	1.81 (0.35–9.35)	0.480
**Tumor Grade**						
G1	Ref.		Ref.		Ref.	
G2	18.76 (4.11–85.49)	<0.001	9.05 (0.63–129.2)	0.104	11.96 (1.55–92.4)	0.017
G3	10 (2.59–38.62)	0.001	14.35 (1.31–157.35)	0.029	6.95 (1.04–46.35)	0.045
**Histologic Subtype**		0.307		0.607		
**Wound complication CD ≥ 2**						
Yes	3.83 (1.24–11.85)	0.02	3.4 (0.66–17.43)	0.142	5.31 (1.1–25.62)	0.037
No	Ref.		Ref.		Ref.	
**Other complication CD ≥ 2**						
Yes	1.15 (0.35–3.77)	0.822	1.22 (0.18–8.35)	0.839	2.94 (0.6–14.46)	0.184
No	Ref.		Ref.		Ref.	
**Adjuvant radiotherapy**						
Yes	0.09 (0.03–0.31)	<0.001	1.1 (0.28–4.32)	0.893	0.22 (0.05–1.02)	0.052
No	Ref.		Ref.		Ref.	

*ASA* American Society of Anesthesiologists, *5-y-LRFS* 5-year local recurrence free survival, *5-y-MFS* 5-year metastasis free survival, *5-y-DSS* 5-year disease free survival, *Ref.* Reference.
